# Bi-functional photocatalytic heterostructures combining titania thin films with carbon quantum dots (C-QDs/TiO_2_) for effective elimination of water pollutants

**DOI:** 10.1007/s11356-023-27285-7

**Published:** 2023-05-10

**Authors:** Pinelopi P. Falara, Islam Ibrahim, Adamantia Zourou, Labrini Sygellou, David Emanuel Sanchez, George Em. Romanos, Lida Givalou, Maria Antoniadou, Michalis K. Arfanis, Changseok Han, Mauricio Terrones, Konstantinos V. Kordatos

**Affiliations:** 1 School of Chemical Engineering, National Technical University of Athens, 9 Iroon Polytechniou St., 15780 Zografou, Athens, Greece; 2 Institute of Nanoscience and Nanotechnology, National Center for Scientific Research “Demokritos”, 15341 Agia Paraskevi, Athens, Greece; 3 Department of Chemistry, Faculty of Science, Al-Azhar University, Nasr City, Cairo, 11884 Egypt; 4 Foundation of Research and Technology Hellas, Institute of Chemical Engineering and High Temperature Chemical Processes (FORTH/ICE-HT), POB 1414, GR-26504 Patras, Greece; 5 Department of Materials Science and Engineering and Center for 2-Dimensional and Layered Materials, The Pennsylvania State University, University Park, PA 16802 USA; 6 Department of Chemical Engineering, University of Western Macedonia, ZEP, 50100 Kozani, Greece; 7 Department of Environmental Engineering, INHA University, 100 Inha-ro, Michuhol-gu, Incheon, 22212 South Korea; 8 Program in Environmental & Polymer Engineering, Graduate School of INHA University, 100 Inha-ro, Michuhol-gu, Incheon, 22212 South Korea; 9 Department of Physics, The Pennsylvania State University, University Park, Pennsylvania 16802 USA; 10 Department of Chemistry and Materials Research Institute, The Pennsylvania State University, University Park, PA 16802 USA

**Keywords:** Synthesis, Carbon quantum dots, C-QDs/TiO_2_ composite, Organic-inorganic pollutants, Photocatalytic degradation, Reusability experiments

## Abstract

In this study, carbon quantum dots (C-QDs), prepared via hydrothermal-microwave procedures, were successfully combined with nanostructured titania (TiO_2_). The photocatalytic oxidation/reduction activity of the C-QDs/TiO_2_ composite films was evaluated in the decomposition of organic-inorganic contaminants from aqueous solutions under UV illumination. Physicochemical characterizations were applied to investigate the crystal structure of the carbon quantum dots and the composites. It was found that the prepared C-QDs/TiO_2_ composites had great contribution to the photocatalytic reduction of hexavalent chromium (Cr^+6^) species and 4-Nitrophenol (PNP) as well as to the photocatalytic oxidation of methylene blue (MB) and Rhodamine B (RhB) dyes. The mechanism of the photocatalytic reaction was studied with trapping experiments, revealing that the electron (e^−^) radical species were powerfully supported for the photocatalytic reduction of Cr^+6^ and PNP and the holes (*h*^*+*^) are the main active species for the photocatalytic oxidation reactions.

## Introduction

Titanium dioxide (TiO_2_)-based photocatalysis is a well-established advanced oxidation process (AOP), which is addressed in numerous environmental remediation and health protection applications, including decomposition of organic contaminants in the aquatic environment or the air (Han et al. 2009; Wang et al. 2014; Stylidi et al. 2004; Ibrahim et al. 2016), inactivation of pathogenic microorganisms (Wang et al. 2014; Reddy et al. 2017; Demirel et al. 2018), and elimination of cancer cells (Hariharan et al. 2020; Stefanou et al. 2010; Lagopati et al. 2014). Nowadays, the usage of TiO_2_ photocatalysts is also explored in advanced reduction processes (ARPs) and the respective technologies (ARTs) in order to verify their applicability against a wider range of pollutants (Athanasekou et al. 2018; Ibrahim et al. 2019; Ibrahim et al. 2020a,b). Moreover, significant research efforts are targeting in the expansion of light absorption region of TiO_2_ above the UV range of solar spectrum through the semiconductor’s energy gap decrement (He et al. 2018; Xu et al. 2018; Reza et al. 2017; Shayegan et al. 2018; Humayun et al. 2018) and the separation rates enhancement of the photogenerated charge carriers (Chen et al. 2002; Zhou et al. 2012). In addition, morphological characteristics and crystallinity properties of the catalyst could affect drastically their specific surface area and physicochemical properties, which directly associated with their adsorption capacity and photocatalytic activity (Ibrahim et al. 2020a,b). In these regards, the surface engineering, the doping with various elements, and the modification with other nanostructured materials have been extensively conducted to deal with the aforementioned challenges for TiO_2_.

A successful approach of titania modification is the fabrication of composites with carbon-based nanomaterials (Zhou et al. 2010; Lee et al. 2019) such as carbon nanotubes (CNTs) (Yu et al. 2005; Murgolo et al. 2015; Zhu et al. 2018; Samy et al. 2020; Martinez et al. 2011), graphene (Qiu et al. 2015; Zhao et al. 2012; Li et al. 2016), graphitic carbon nitride (g-C_3_N_4_) (Ji et al. 2020; Zhang et al. 2016) or more recently with carbon quantum dots (C-QDs) (Zhou et al. 2012). In all cases, the carbonic materials are well-combined with TiO_2_, leading to significant improvement of the photocatalytic degradation of emerging pollutants, through the enhancement of surface adsorption and the photo-charge carriers’ separation. C-QD discovered by Xu et al. (2004) are considered as very efficient and promising candidates for photocatalytic purification of water because they are zero-dimensional nanomaterials (0D), synthesized by low-cost and simple synthetic techniques, and presenting good electrons accept and transfer properties, high water solubility, and low toxicity. Primarily, C-QDs are used to form composite materials with TiO_2_ or other semiconductors (Ke et al. 2017; Zhang et al. 2019; Zhao et al. 2020; Khare et al. 2018; Kumar et al. 2017; Al Marzouqi et al. 2019), with the intention of the photocatalytic activity improvement. The enhancement of the photocatalytic performance is attributed to the acceleration of interfacial charge transfer process, the suppression of recombination rates of charge carriers, and the widening of visible absorbance. Additionally, C-QDs could play the role of electron reservoirs to trap photoexcited electrons, which improves charge separation efficiencies. These abilities can overcome the limitations of photocatalysis and enhance the activity of the photocatalyst (Zhang et al. 2016; Molaei 2020; Li et al. 2015; Dhenadhayalan et al. 2020; Yu et al. 2014; Araujo et al. 2016; Falara et al. 2022). Furthermore, C-QDs provide plenty of active sites due to their high surface area, thus are capable of improving the adsorption ability (Akbar et al. 2021, Song et al. 2022).

In this work, carbon quantum dots/titanium dioxide (C-QDs/TiO_2_) nanocomposite has been fabricated for photocatalytic applications. First, the structural, morphological, electrochemical, and spectroscopic properties of the C-QDs/TiO_2_ catalyst were studied using Micro-Raman, X-ray powder diffraction (XRPD), ultraviolet–visible spectroscopy (UV-Vis), Fourier transform infrared spectroscopy (FTIR), photoluminescence spectroscopy (PL), X-ray photoelectron spectroscopy (XPS), scanning electron microscopy (SEM), and scanning transmission electron microscopy (STEM). Then, the photocatalytic activity of the C-QDs/TiO_2_ composite was evaluated in the oxidative degradation of Rhodamine B (RhB) and methylene blue (MB) organic dyes under UV light illumination and in the photocatalytic reduction of 4-nitrophenol (PNP) and hexavalent chromium (Cr^6+^) water contaminants. The reason we decide to study the degradation of these specific substances is their widespread use in industry resulting in the uncontrolled discharge into the environment, which makes them an important factor in environmental pollution. The C-QDs/TiO_2_ catalyst’s performance was compared with the reference TiO_2_ photocatalyst and it was proved that the novel C-QDs/TiO_2_ material exhibits highly improved photocatalytic properties.

## Experimental

### Materials

All chemicals with analytical grade were used without further purification. Titanium (IV) isopropoxide (C_12_H_28_O_4_Ti, ≥ 98%), potassium bromate (KBrO_3_, ≥ 98%), potassium iodide (KI, ≥ 98%), benzoquinone (C_6_H_4_O_2_, ≥ 98%), isopropyl alcohol (C_3_H_8_O, ≥ 98%), urea (CH_4_N_2_O, ≥ 98%), and absolute ethanol (C_2_H_5_OH, ≥ 99%) were purchased from Acros-Organics. Glacial acetic acid (CH_3_COOH, ≥ 98%), 1, 5- Di-phenylcarbazide (C_13_H_14_N_4_O, ≥ 98%), Triton X-100 (C_16_H_26_O_2_, ≥ 99%), terpineol (C_10_H_18_O, ≥ 98%), and cellulose ethane (C_34_H_66_O_24_, ≥ 99%) were all obtained from Sigma-Aldrich. Methylene blue (C_16_H_18_C_l_N_3_S, ≥ 98%), sodium hydroxide (NaOH, ≥ 99%), and potassium dichromate (K_2_Cr_2_O_7_, 98%) were supplied from Fluka, while Rhodamine B (C_28_H_30_N_2_O_3_, ≥ 98%), sodium sulfate (Na_2_SO_4_, ≥ 99%), and 4-nitrophenol (C_6_H_5_NO_3_, ≥ 98%) were obtained from Merck. Hydrochloric acid (HCl, 37%) and absolute acetone (C_3_H_6_O, ≥ 99%) were purchased from Riedel-de Haen. Potassium hydroxide (KOH, ≥ 99%), Nafion perfluorinated (C_7_HF_13_O_5_S.C_2_F_4_, ≥ 98%), and potassium chloride (KCl, ≥ 99%) were obtained from Chem-Lab. The reference electrode Ag/AgCl with electrolyte concentration C_KCl_ = 3 mol/L was supplied from Metrohm. Finally, microscope slides were purchased from Fisher Scientific, while deionized (DI) water was used throughout.

### Preparation of the reference and composite photocatalytic films

#### Preparation of reference TiO_2_ films

The mesoporous photocatalytic films of the commercial Degussa (Evonik) P25 powder were deposited onto conductive fluorine-doped tin oxide (FTO) glass substrates. Primarily, a compact layer of TiO_2_ between the FTO and the mesoporous film was prepared using a sol-gel technique, as follows: initially, a mixture of 3.6 mL titanium (IV) isopropoxide, 6.8 mL CH_3_COOH, and 38.0 mL ethanol (EtOH) were mixed and stirred for about 5 min. Then, 7.0 g of Triton X-100 was gradually added to the above mixture. Finally, the deposition held by immersing the FTO substrates vertically into the sol solution for a few seconds and calcinated at 550 °C for 30 min. This procedure was repeated 4 times, aiming to create a thick titania compact layer, which will improve the mesoporous layer adhesion and electroconnectivity with the FTO substrate (Givalou et al. 2020).

For the mesoporous layers, a viscous paste was first prepared using the commercial Degussa (Evonik) P25 powder. Briefly, 3.0 g of P25 powder was grinded in a mortar by adding progressively a total of 0.5 mL of CH_3_COOH and 1.5 mL of DI water. Then, 17.5 mL of EtOH were gradually added with continuous grinding. The above mixture was transferred to a round-bottomed flask, with the addition of 50 mL EtOH and was stirred in an ultrasonic bath. After that, 10.0 g of terpineol and 2.8 g of 10% v/v ethyl cellulose ethane solution were added, followed by stirring in an ultrasonic bath. A rotary evaporator was used to perform the evaporation of the ethanol at 50 °C. For the reference sample, the solution mentioned above was first dispersed in EtOH in a 1:1 ratio, and then the deposition was performed on the compact layer TiO_2_ by the doctor blade technique, followed by annealing at 550 °C for 30 min (Givalou et al. 2020). This process was repeated 4 times, so as a thick titania mesoporous layer was created on the compact layer substrate surface, with increased available photocatalyst volume. This reference titania film was named as T.

### Synthesis of carbon quantum dots (C-QDs) and the composite TiO_2_/C-QDs films

The C-QDs were prepared with a hydrothermal synthetic route. In this method, a ratio of citric acid: urea (as a source of fuel for annealing) 1:1 was added to 10 mL of DI water. The mixture was stirred vigorously for about 10 min and then was transferred to a Teflon-lined autoclave. Then, the autoclave was placed in a microwave radiation reaction system. The parameters of the experimental process were defined as follows: pressure up to 40 bar, microwave radiation energy 800 Watt, temperature 200 °C, and time interval 15 min (Qu et al. 2012). After natural cooling, the resulting solution was centrifuged at 6000 rpm for 30 min and filtrated through a plain filter paper in order to separate the C-QDs solution from by-products. Thus, the precipitate was removed, while the supernatant solution of C-QDs was mixed with acetone. The obtained sediment was dissolved in EtOH, stored in a closed container, and was named CD.

The final C-QDs solution was mixed with the commercial titania P25 solution in a 1:1 ratio to prepare mesoporous photocatalytic films of the composite TiO_2_/C-QDs materials, named as TCD. The exact coating process remained the same, as previously. In that point, it should be mentioned that the dilution of the reference precursor solution in 2.1.1 was performed in order to be equivalent with the TiO_2_/C-QDs precursor solution and prepare equivalent films for both reference and composite materials.

### Nanocomposite characterization

X-ray powder diffraction (XRPD) analysis was performed using a Siemens D-500 diffractometer, which operates in Bragg- Brentano geometry with Cu K_α1_ (*λ* = 1.5406 Å) and Cu K_α2_ (*λ* = 1.5444 Å) radiation. Data were collected over the angular range from 10 to 80°, counting for 3 s at each step of 0.02° in the detector position.

Micro-Raman spectra were measured in the backscattering configuration on a Renishaw via Reflex microscope, using diode lasers emitting at *λ* = 514.5 nm, as excitation sources. The laser beam was focused onto the samples by means of a 50× objective on a Leica DMLM optical microscope, while the laser power density was kept below 1.5 mW μm^−2^.

IR spectra were collected on a Thermo Scientific Nicolet 6700 FTIR with N_2_ purging system. Spectra were acquired using a transmission cell with a KBr pellet. A total of 32 scans were averaged for each sample and the resolution was 4 cm^−1^. The spectra were obtained against a single beam spectrum of the pure KBr Pellet. Data were collected in the range of 4000–400 cm^−1^.

The optical properties of the samples were analyzed by UV–vis diffuse reflectance spectroscopy, using a Hitachi 3010 spectrophotometer equipped with a 60-mm-diameter integrating sphere and BaSO_4_ was used as a reference. The absorption data were expressed in with Kubelka–Munk units by using the respective equation (F(R)).

Fluorescence spectrum was obtained using 4.5-mL volume cuvette with a Perkin-Elmer LS 45 Luminescence Spectrometer. The sample was excited at 330 nm that corresponds to its maximum absorption. The slit width was set at 10 nm. Scan speed was adjusted to 480 nm min^−1^. The measurement was carried out at room temperature. The sample was freshly prepared just before measurement.

Scanning electron microscopy (SEM) was conducted on a Thermo Scientific Verios G4 operating at 2 kV in secondary electron (SE), while scanning transmission electron microscopy (STEM) images were acquired using a FEI Talos F200X microscope operating at 200 kV. The powder was dispersed in 1 mL of DI water and sonicated for 10 min. A drop was placed on a lacey carbon copper grid.

The pore structural properties of the prepared materials were analyzed by liquid nitrogen (LN_2_) adsorption-desorption isotherms at 77 K using an automated volumetric system (AUTOSORB-1 Quantachrome Instruments). The volumetric method was used to calculate the adsorbed amount of nitrogen. Prior to each measurement, the samples were outgassed at 180 °C for 24–48 h, under high vacuum achieved by a turbomolecular pump. The specific surface area was obtained by Brunauer-Emmett-Teller (BET) method and the size distribution of the pores was calculated by the Barrett-Joyner-Halenda (BJH) method based on a modified Kelvin equation.

For XPS measurements, the photoemission experiments were carried out in an ultra-high vacuum system (UHV) equipped with a SPECS DLD 100 hemispherical energy analyzer, X-ray gun, and UV source (model UVS 10/35) for XPS/UPS measurements. Unmonochromatized MgK_α_ line at 1253.6 eV and analyzer pass energy of 15 eV (giving a full width at half maximum (FWHM) of 0.85 eV for the Ag3d5/2 peak) was used for the XPS measurements. The XPS core level spectra were analyzed using a fitting routine, which can decompose each spectrum into individual mixed Gaussian-Lorentzian peaks after a Shirley background subtraction. Errors in our quantitative data are found in the range of ~10%, (peak areas), while the accuracy for BEs assignments is ~0.1 eV. UPS measurements were recorded using HeI irradiation (hν = 21.22 eV) and the analyzer was working at the constant retarding ratio (CRR) mode, with CRR = 3. From the UPS spectra, the work function (WF) and the valence band maximum cutoff (VBM) were directly measured by linear extrapolation of the high and low energy cutoffs to the baseline and determining their intersections with the binding energy axis. For the WF determination, a bias of −12.30 V was applied to the sample in order to avoid interference of the spectrometer threshold in the UPS spectra.

Electrochemical measurements were performed via an Autolab potentiostat (PGSTAT-302N), Photocurrent−Time (I−t) characteristics were obtained at open circuit potential and the illuminated (active) area was 1 cm^2^, working in a standard 3-electrode system. A platinum foil (Pt) and a silver/silver chloride (Ag/AgCl) with electrolyte concentration C_KCl_ = 3 mol/L were used as counter and reference electrode respectively. The working electrode was prepared as follows: FTO transparent conductive glass electrodes (7 ohms cm^−2^, Pilkington) were first washed with soap (2% Hellmanex in water), deionized water, and ethanol. For the preparation of the photoelectrode, 5 mg of the photocatalyst were dispersed in 25 μL Nafion perfluorinated solution, 145 μL 3D water, and 84 μL absolute ethanol, followed by ultrasonication for 2 h that resulted in a uniform suspension. The prepared suspension was doctor-bladed on the FTO electrode to form a uniform 1 cm^2^ active area. The sample was then annealed at 450 °C. The electrolyte used for the electrochemical measurements was 0.5 M sodium sulfate solution (Na_2_SO_4_) and the illumination source was simulated solar light (1 sun, 1000 Wm^−2^) from a Xenon 300 W source in combination with AM 1.5 G optical filters. Impedance measurements (EIS) were performed on complete cells under 1-sun illumination conditions by applying a forward bias at Voc conditions, using the PG-STAT-30 potentiostat and its built-in frequency response analyzer (FRA).

### Evaluation of the photocatalytic oxidation/reduction activity

The photocatalytic performance of the reference and composite films was evaluated by studying the photocatalytic reduction of two toxic compounds, Cr^6+^ (5 mg L^−1^) and PNP (10 mg L^−1^), and the photocatalytic oxidation of two water-soluble organic dyes, MB (5 mg L^−1^) and RhB (4 mg L^−1^). The films were first immersed in 10 mL aqueous solution of the respective pollutant, until adsorption-desorption was achieved after 1 h. Next, UV-A illumination was employed for 2 h by 4 UV-A Sylvania lamps (350–390 nm, 0.5 mW cm^−1^) onto a lab-made photocatalytic reactor (Arfanis et al. 2017). Every 30 min, the pollutants’ characteristic absorption peak was measured with the UV/Vis spectrophotometer, with the purpose of defining the reaction kinetic (Papoulis et al. 2018). In the case of chromium, a colorimetric technique based on the diphenylcarbazide (DCP) metal ion indicator was applied (Ibrahim et al. 2020a,b, Bortolotto et al. 2022). The photocatalytic reduction of Cr^6+^ consumes protons and as a result, Cr^6+^ can be reduced effectively only in an acidic medium. The pH is a critical parameter for hexavalent chromium reduction in TiO_2_ photocatalysis (Djellabi et al. 2022, Djellabi et al. 2020). For this reason, 0.1 mL of H_2_SO_4_ (0.2 mol/L) were added in the Cr^+6^ solution. In parallel, control experiments under UV-A were performed in the absence of photocatalysts. No concentration alterations occurred for Cr^6+^ and PNP through photolytic reduction reactions or photolytic oxidation reactions for MB and RhB (data not shown).

In order to distinguish the photocatalytic oxidation-reduction mechanism of MB, RhB, Cr^+6^, and PNP pollutants under UV light illumination, trapping experiments were performed for the most efficient catalysts (Antoniadou et al. 2019). In these experiments, four different scavengers were selected and added into the pollutants’ solutions: benzoquinone (BQ—1 mM) as O_2_^−^ quencher, potassium bromate (KBrO_3_—10 mM) as e^−^ quencher, potassium iodide (KI—10 mM) as h^+^ quencher and isopropyl alcohol (IPA—10 mM) as OH quencher. By verifying the scavengers’ effect on the photocatalytic efficiency, it was elucidated which species were contributing in the respective photocatalytic processes.

## Results and discussion

### Characteristics of carbon quantum dots

**Fig. 1:**
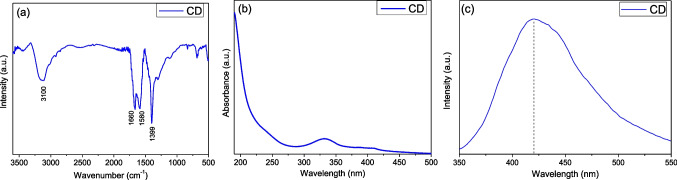
Comparative FT-IR spectrum (**a**), UV-Vis absorption spectrum (**b**), and PL spectrum under 330 nm excitation wavelength (**c**) of C-QD material

The results of characterization of C-QDs material using the FT-IR technique, which was used to identify their chemical structure, are presented in Fig. 1a. More specifically, in FT-IR spectrum, the vibration at 3100 cm^−1^ is attributed to of N–H bond stretching vibrations, while a weak peak at 3400 cm^−1^ is due to the stretching vibration of the O–H bonds (Ke et al. 2017). In addition, the characteristic peak of the sp^2^ bonds C=C is observed at 1580 cm^−1^, which is arising from the graphitic regions of the core of C-QDs (Zhang et al. 2019). Furthermore, two peaks at 1660 cm^−1^ and 1398 cm^−1^ are attributed according to the literature, to the presence of C=O and C–H bonds, respectively (Tan et al. 2013; Atchudan et al. 2019; Chang et al. 2016). Last, the footprint vibrations at the lower wavenumber region (from 1300 to 640 cm^−1^) are implying additional stretching and bending vibrations of C–O and C–H bonds, respectively (Al Marzouqi et al. 2019).

As shown in Fig. 1b, the UV-Vis spectrum of C-QDs presents a distinct absorption peak at wavelength equal to 330 nm and an intense shoulder in the UV part of solar spectrum (below 220 nm). According to the literature, this absorption peak is attributed to n→π* electronic transitions corresponding to the C=O/C=N bonds, which are probably located on the surface of C-QDs (Ludmerczki et al. 2019; Devi et al. 2018; Niu et al. 2015; Zhu et al. 2013; Wu et al. 2017). Concerning the absorption shoulder in the UV region, it is defined as typical C=C transition bands of graphitic core (Givalou et al. 2020; Qu et al. 2012).

Moreover, the optical properties of C-QDs were studied by PL spectroscopy (Fig. 1c). It is noted that the PL spectrum was obtained by selecting an excitation source emitting at 330 nm, close to the maximum absorption band in the respective UV-Vis spectrum, while the sample was diluted appropriately, with the aim of avoiding any instrumental measurement limitations. A characteristic and intense fluorescence, corresponding to a wavelength value equal to 420 nm, was observed. The origin of the PL phenomenon in quantum carbon dots has not yet been totally understood; however, it could be related with the multiple active optical centers, such as the graphite core of quantum carbon dots and/or the functional groups on their surface (Arfanis et al. 2017).

### Composite characterization

#### Morphology characterization

**Fig. 2:**
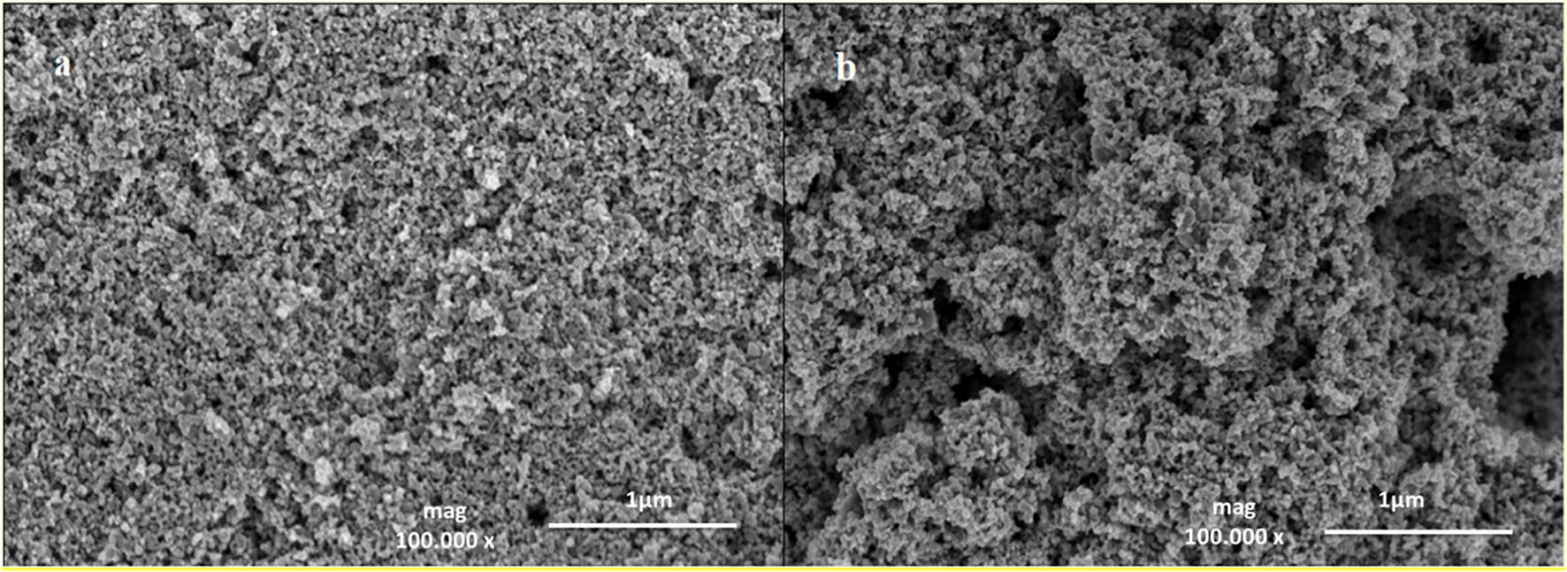
SEM images of T (**a**) and TCD nanocomposite (**b**)

Figures 2a and b present the SEM images of the T and TCD composite, respectively, in which the nanoparticles intertwine with each other to create a porous spherical morphology because of the formation of TiO_2_ clusters. In Fig. 2b, the C-QDs are attached on the surface of TiO_2_. Nevertheless, due to the small particle size of the C-QDs and the agglomeration existing in TCD nanohybrid, a clear observation using SEM is not achievable and it is difficult to obtain the exact morphology of it.

**Fig. 3:**
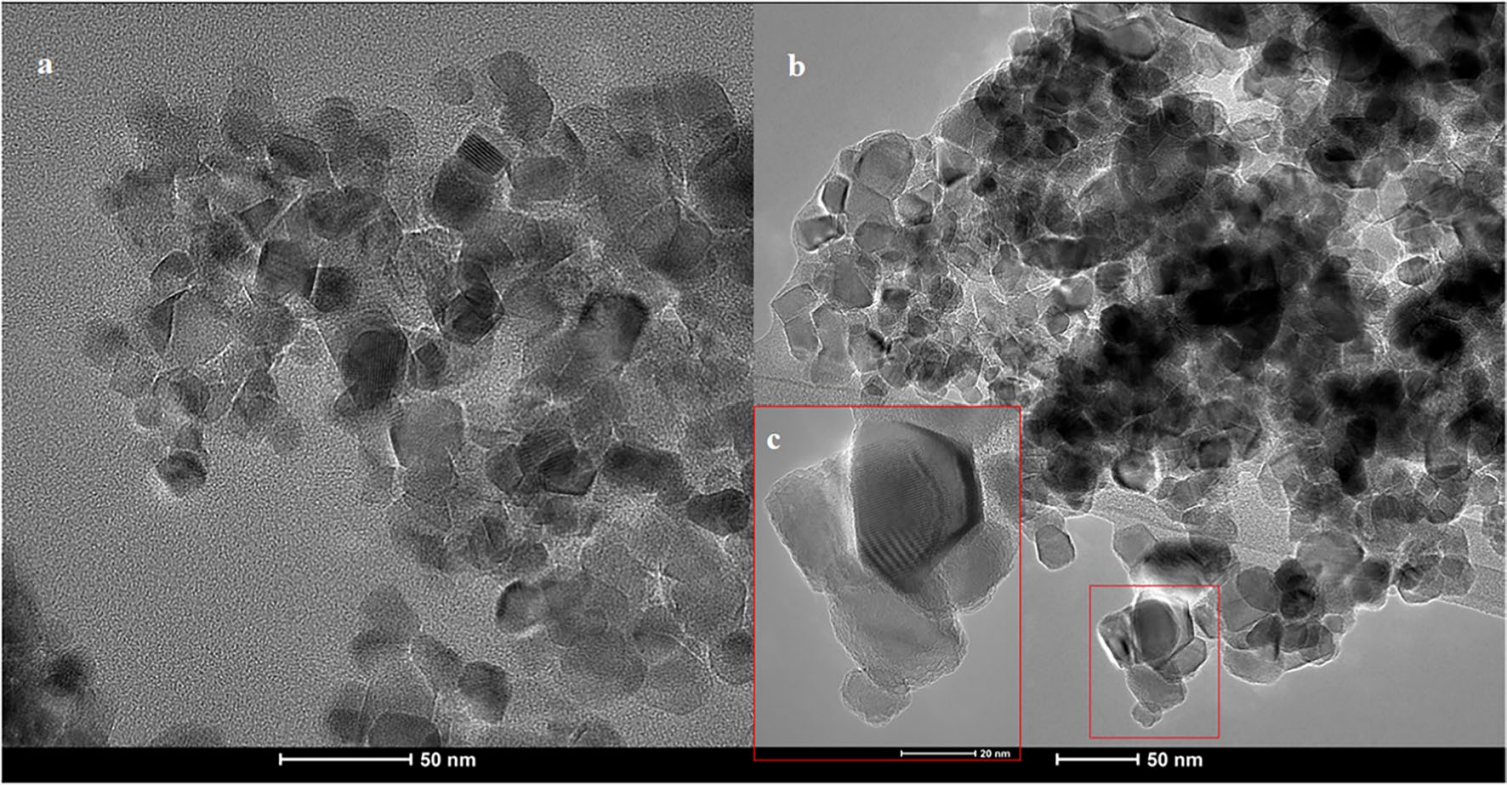
TEM images of T (**a**) and TCD nanocomposite (**b** and **c**)

Additionally, the particle size and morphology of the T and TCD materials were characterized by scanning transmission electron microscopy (S/TEM). TEM images in Fig. 3 confirm the materials’ nanostructure and it can be estimated that the average size of the nanoparticles is about 20 nm. It can be seen that the TiO_2_ nanoparticles occupy most of the available surface area which provides a high loading of C-QDs in the TCD nanohybrid material. Thus, TEM images of TCD (Fig. 3b and c) demonstrate that TiO_2_ nanoparticles are slightly rod-shaped and C-QDs are well-dispersed on their surface.

### Porosimetry measurements

**Fig. 4:**
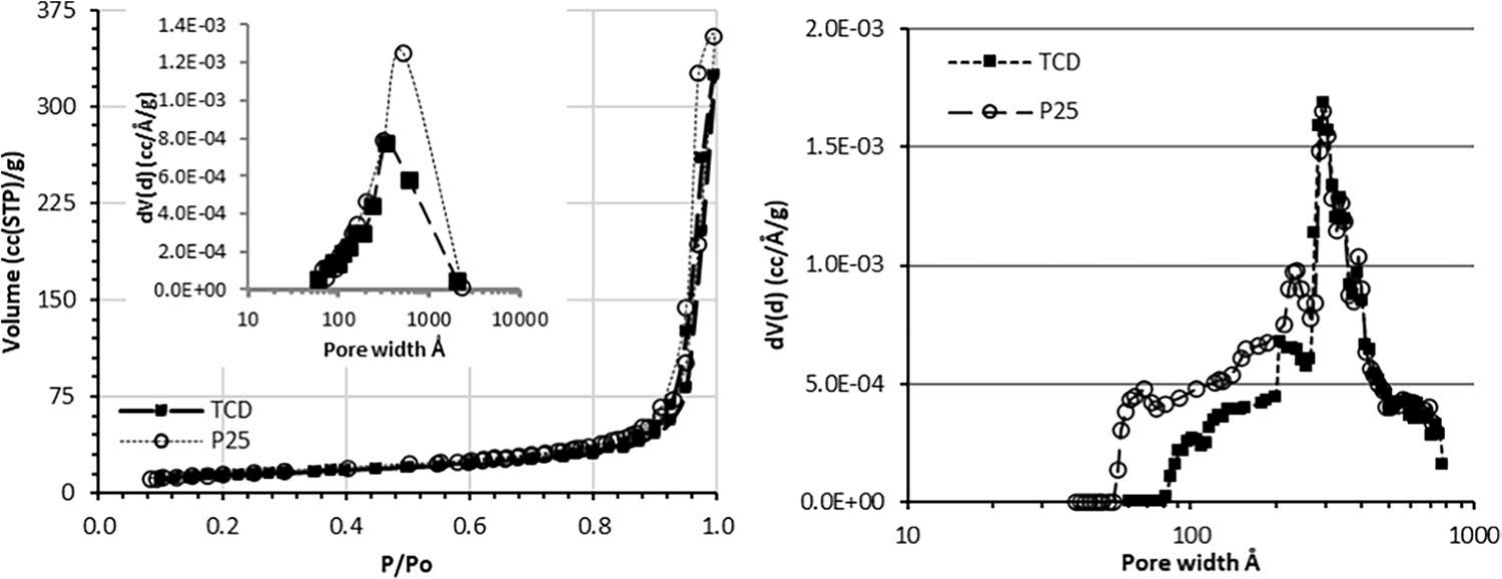
Comparison of the N_2_ adsorption-desorption isotherms between the nanocomposite (TCD) and reference (P25) samples (inset: pore size distributions obtained from the desorption branch with the BJH method) (**a**) and pore size distribution of the prepared TCD nanocomposite and the reference P25 material, derived with the NLDFT method (**b**)

The N_2_ adsorption-desorption isotherm of the TCD composite at 77 K in comparison to that of the reference TiO_2_ P25 sample is presented in Fig. 4a. Both isotherms are of type II, which according to the IUPAC classification is characteristic for non-porous or macroporous adsorbents with strong adsorbate-adsorbent interactions. It can be also observed that the isotherms are not fully reversible at the high relative pressures (P/Po) region. Hence, the occurrence of a slight hysteresis loop is indicative for the existence of large mesopores (20–50 nm) which correspond to the interstitial space between the closely packed TiO_2_ nanoparticles. Α general remark coming out by comparing the isotherms and the respective pore size distributions (inset Fig. 4a and b) of the TCD and P25 samples is that the presence of C-QDs in the nanocomposite (TCD) does not significantly affect the textural and pore structural characteristics of the reference photocatalytic material (P25), as expected due to the very small size of C-QDs (~2–5 nm). Indeed, the BET surface area of TCD was calculated at 48.6 m^2^/g when the respective one for P25 was 54.5 m^2^/g. The slight decrease can be attributed to partial inhibition of the accessibility of N_2_ in the interstitial space between the TiO_2_ nanoparticles due to the deposition of C-QDs, some of them nesting close to the narrower pore openings.

The pore size distribution curves (Fig. 4b) calculated with the NLDFT method (Calc. Model: N_2_ at 77 K on silica (Cylindr. Pore NLDFT adsorption branch model) display a maximum distribution of pores diameter at 300 Å for both samples, while the inclusion of C-QDs is responsible for the blockage of a small fraction of the P25 pores with a size of 40–70 Å. As a result, the total pore volume is reduced from 0.55 cc/g in the P25 sample to 0.5 cc/g in the nanocomposite (Fig. 4a). It is therefore worth to mention that since the inclusion of C-QDs has not any significant effect on the textural and pore structural features of the P25 sample, the experimentally verified higher adsorptivity of the nanocomposite C-QDs/TiO_2_ as compared to the pristine TiO_2_, can only be attributed to the higher affinity of C-QDs for the pollutants under study.

### Structural characterization

**Fig. 5:**
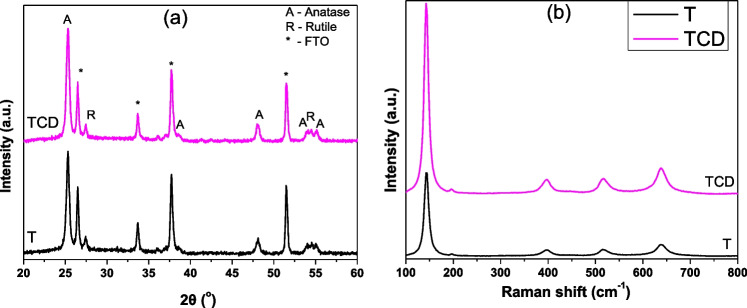
Diffraction patterns of C-QDs modified TiO_2_ film and the respective reference film (**a**) and Raman spectra of the synthesized materials films (**b**)

The X-ray powder diffraction patterns of the synthesized photocatalysts are shown in Fig. 5a. In particular, the characteristic diffraction peaks of anatase polymorph were observed at 25.36°, 37.76°, 48.03°, 53.9°, and 55.01°, which are corresponding to the (101), (004), (200), (105), and (211) crystal planes of the tetragonal anatase phase (JPDS Card No.21-1272). The (110) and (211) crystal planes of rutile co-exist at 27.37° and 54.05° (JCPDS Card No.21-1276), respectively, which is a typical outcome for nanostructured TiO_2_ materials with the commercial Evonik P25 (Kontos et al. 2005). Concerning the carbon quantum dots diffractions, the characteristic signal at 13° for carbon dots or at ~26° for carbon materials was not evident (Liu et al. 2014; Cheng et al. 2017), implying their amorphous nature, their quantum-sized dimensions, their low content, and their uniform and high dispersion on the TiO_2_ film. Apart from these, the addition of the quantum dots did not alter the weight fraction of anatase and rutile, or the TiO_2_ crystal size (Arfanis et al. 2019). It is also mentioned that extra diffractions from the FTO substrate were also present (JPDS 46-1088, noted with asterisk in the figures).

The crystallinity study with the micro-Raman vibrational spectroscopy was in line with XRD results (Fig. 5b). The six Raman bands of anatase were clearly revealed for all samples at 144 (Ε_g_, symmetrical stretching vibration of O–Ti–O), 196 (Ε_g_, symmetrical stretching vibration of O–Ti–O), 396 (B_1g_, symmetrical bending vibration of O–Ti–O), double peak at 516 cm^−1^ (B_g_ and Α_1g_, symmetrical and antisymmetrical bending vibration of O–Ti–O), and 639 cm^−1^ (Ε_1g_, symmetrical stretching vibration of O–Ti–O) (Arfanis et al. 2017). The respective vibrations of rutile polymorph were hardly observed at 446 (E_g_) and 616 cm^−1^ (A_1g_), as the anatase modes prevail over the rutile bands. No signal from the characteristic D and G bands for carbonic materials was detected for the carbon quantum dots modified samples at ~1350 and ~1609 cm^−1^ (Giannouri et al. 2016), suggesting the small size of the carbon dots and their uniform dispersion on titania.

### Optoelectronic properties

**Fig. 6:**
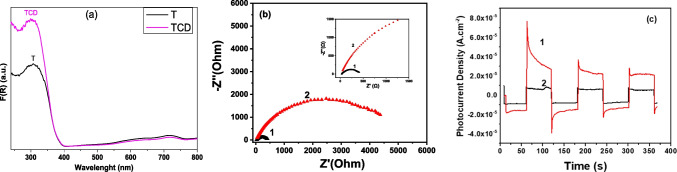
Absorption spectra in Kubelka-Munk for references and C-QDs modified materials (**a**), Nyquist plots for photoelectrodes based on C-QDs/TiO_2_ (1) and TiO_2_ (2) materials (**b**), and corresponding on-off photocurrent density-time curves obtained at Voc in 0.5 M Na_2_SO_4_ (**c**). Electrode active area: 1 cm^2^

Light absorption properties of the pristine and the C-QDs modified TiO_2_ films, expressed in Kubelka-Munk units, were examined (Fig. 6a). Pristine and modified films presented a typical spectrum for titania nanomaterials, as they absorb till the intrinsic band gap of TiO_2_ at around 385 nm. Interestingly, the C-QDs modified films absorb stronger than the bare TiO_2_ films in the UV part of the solar spectrum, showing clearly that the C-QDs are participating in this enhancement. Nevertheless, the absorption was not extended to the visible region of the solar spectrum, indicating that no surface chemical bonding is expected between C-QDs and TiO_2_ through carbon doping (Sharma et al. 2018).

The photoelectrochemical properties of the materials deposited on FTO electrodes were explored in 0.5 M Na_2_SO_4_ under 1-sun illumination conditions (A.M. 1.5 G). Figure 6b presents the on-off photocurrent-time (I-t) curves and confirms that C-QDs/TiO_2_ clearly outperforms the reference titania sample. Such an excellent photoelectric response (about 5 times higher than that of TiO_2_) is attributed to the presence of the carbon quantum dots in the nanocomposite material, which results in enhanced light absorption and facilitates (promotes) the efficient separation of the photogenerated charge carriers (electrons and holes) (Ibrahim et al. 2020a,b). Electrochemical impedance spectroscopy (EIS) measurements performed under 1-sun provide an additional proof on the beneficial role of the carbon dots. Figure 6c shows the Nyquist plots of the samples extracted from EIS data, where the semi-circle diameter for the C-QDs/TiO_2_ electrode is 646 Ohm, significantly smaller than the diameter of the pure titania electrode (5060 Ohm). This corresponds to a lower charge transfer resistance and demonstrates more efficient charge separation and/or higher interfacial charge transfer ability for the carbon dot-containing material, in agreement with the observed enhancement in photocatalytic performance.

### XPS measurements

**Fig. 7:**
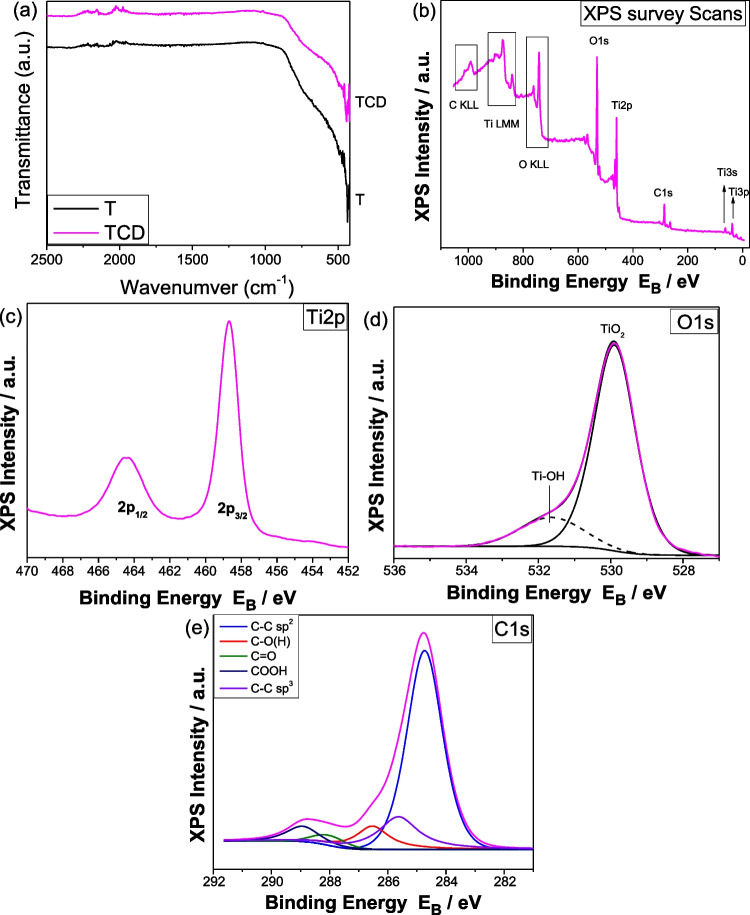
XPS survey scans (**a**), Ti2p XPS peak (**b**), deconvoluted O1s XPS peak (**c**), deconvoluted C1s XPS peak (**d**) of TCD material

The functional groups of the film’s nanocomposites were recognized by using FTIR spectroscopy and shown in Fig. 7a, both for reference and composite materials. For all films, two wide peaks from 880–430 cm^−1^ are depicted, which are related with Ti–O and Ti–O–Ti bonding of titanium dioxide (Zhang et al. 2020). The successful loading of carbon quantum dots over TiO_2_ film was confirmed by the broad bands of TCD film in this region, compared to the reference film T, which is attributed as Ti–O–C vibrations (Devi et al. 2018).

The XPS survey scans in Fig. 7b show the presence of Ti, C, and O atoms on the samples surface. The detailed Ti2p spectra consist of a doublet with a spin-orbit splitting of 5.7 eV and a binding energy of Ti2p3/2 at 458.8 eV assigned to TiO_2_ chemical state (Fig. 7c). Figure 7d shows the deconvoluted O1s peak for TCD, which consists of two components at 529.8 eV due to TiO_2_ and at 531.6 eV due to Ti–OH bonds (Arfanis et al. 2019). Figure 7e shows the C1s peak analyzed into five components assigned to C–C bonds with sp^2^ and sp^3^ hybridization (284.7 eV and 285.6 eV respectively), hydroxyls and/or epoxides (C–O, at 286.5 eV), carbonyls (C=O, at 288.5 eV), and carboxyls (COOH, at 289.3 eV) (Sygellou et al. 2016). Table 1 shows the % oxygen components concentration and carbon components concentration derived from the O1s and C1s XPS peak deconvolution. Also, it shows the % relative atomic concentration is determined from the peak areas of Ti2p, O1s, and C1s dividing them with the corresponding relative sensitivity factors and analyzer transmission function (Zhang et al. 2017).

**Table 1:** Carbon and oxygen components concentration derived from the C1s and O1s peak deconvolution and % relative atomic concentration derived from the XPS peak areas

Carbon components					Oxygen components		% relative atomic concentration		
C-C sp^2^	C-C sp^3^	C-O	C=O	COOH	TiO_2_	Ti-OH	Ti	O	C
65.2	16.8	10.1	2.9	5.0	83.1	16.9	19.7	51.6	28.7

**Fig. 8:**
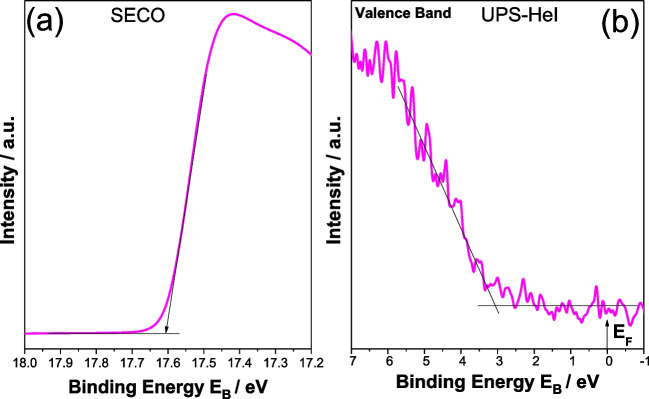
UPS spectrum taken from the surface (**a**), secondary electron cut-off region and valence band region (**b**) of TCD material

Figure 8 illustrates the UPS spectra, taken from sample TCD. The work function (Fig. 8a) can be calculated from the difference between the incident light energy (hν = 21.22 eV) and the energy of the secondary cutoff: WF = hν-E_cutoff_ (Table 2). Figure 8b shows the valence band region and the valence band maximum (VBM) cutoff. The ionization potential is derived from the sum of the WF and the VBM and presented in Table 2 (Hannula et al. 2018).

**Table 2:** Work function, valence band maximum and ionization potential of the TCD sample

Sample	WF/eV	VBM cutoff/eV	IP (± 0.1) eV
TCD	3.6	3.4	7.0

### Oxidation and reduction photocatalytic processes

#### Photocatalytic reduction of hexavalent chromium and 4-nitrophenol

**Fig. 9:**
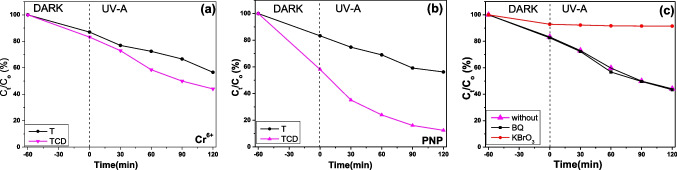
Photocatalytic reduction of Cr^+6^ (**a**) and PNP (**b**) under UV light irradiation using the synthesized photocatalytic films. Effect of scavengers on photocatalytic degradation of Cr^6+^ during the trapping experiments of TCD (**c**)

The photocatalytic reduction activity of the prepared photocatalytic films was evaluated by converting the toxic Cr^6+^ and PNP to less-toxic Cr^3+^ and 4-Amino Phenol (4-AmP), respectively, under UV-A light illumination, as presented in Fig. 9. It is noted that the negative time values in the time axes just correspond to the requisite time for the adsorption-desorption equilibrium under dark conditions. The examination of the graphs and the collected data in Table 3 reveals that the TCD composite enhanced the photocatalytic reduction efficiencies of titanium dioxide towards hexavalent chromium and 4-nitrophenol close to 40% compared with the reference film Τ. In particular, 43.39% of the toxic Cr^6+^ transformed to the less-toxic Cr^3+^ with TCD, while the photocatalytic reduction of PNP reached 87.53%. Langmuir-Hinshelwood kinetics was used to determine the rate constant as follows:

$$\ln \frac{\complement }{\textrm{C}0}=-k.t$$

**Table 3:** Removal efficiency and rate constant of Cr^6+^ and PNP with the synthesized films

Samples	Removal efficiency (%)		Rate constant min^−1^	
	Cr(IV)	PNP	Cr(IV)	PNP
T	43.39	43.76	0.00474	0.00480
TCD	55.97	87.53	0.00683	0.01735

where *k* is the rate constant (min^−1^), *C*_0_ is the initial concentration of the Cr(VI) and PNP, and *t* is the photodegradation time (min) (Wang et al. 2020). The calculated rate constants of the composite against chromium and 4-nitrophenol reduction show a remarkable photocatalytic performance under UV light illumination, in accordance with the photocatalytic removal efficiencies (Table 3). Moreover, there was a noticeable improvement of PNP adsorption onto the composite catalysts before the illumination.

**Fig. 10:**
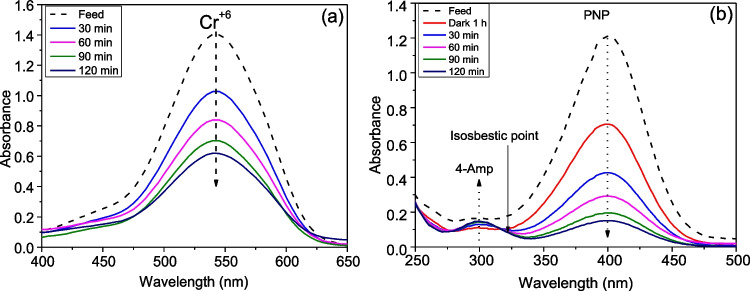
UV–vis absorption spectra through the photocatalytic reduction of Cr^+6^ (**a**) and the reduction spectra of PNP (**b**), respectively

Figure 10 presents the UV–vis absorption spectra of the photocatalytic reduction of both Cr^+6^ and PNP at different reaction times, in the presence of TCD. Figure 10a shows the progressive evolution of hexavalent chromium using TCD nanocomposite film at 30-min intervals. The absorption peak of (VI)-diphenylcarbazide complex, which is located at 542 nm, was rapidly lessened in intensity with the photocatalytic reaction time. In the case of PNP, its absorption peak at 400 nm shows an advanced declining, while a new peak gradually raised at 300 nm, which is related to 4-AmP (Fig. 10b). Interestingly, the existence of isosbestic point approves the formation of only 4-AmP without any intermediate products.

The photocatalytic results imply that C-QDs nanoparticles have a vital role in improving the photocatalytic reduction processes. Possibly, the assistance of the better charge carrier’s separation at the interface TiO_2_–carbon dot could be the reason for the enhanced Cr^6+^ and PNP photocatalysis on the composite films, without excluding the formation of additional superoxide radicals. In order to examine which reactive species, e^−^ or O_2_^●−-^, were responsible for the composite film performance, photocatalytic trap experiments were employed for hexavalent chromium in the presence of BQ and KBrO_3_. As shown in Fig. 9c, chromium reduction efficiency remained unaffected in the presence of the BQ, whereas the photocatalysis was totally hindered when the KI was added in the solution. Based on these results, it is demonstrated that the photoelectrons were mainly responsible for the Cr^6+^ photocatalytic reduction enhancement of the composite films.

#### Photocatalytic oxidation of methylene blue and Rhodamine B dyes

**Fig. 11:**
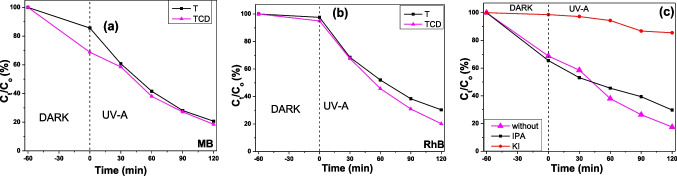
Photocatalytic oxidation of MB (**a**) and RhB (**b**) under UV light irradiation using the synthesized photocatalyst films. Effect of scavengers on photocatalytic degradation of MB during the trapping experiments of TCD (**c**)

The photocatalytic oxidation ability for the reference and composite films was examined with two common test pollutants, the azo-dye methylene blue (Fig. 11a) and the water fluorescent tracer Rhodamine B (Fig. 11b). Even if TCD were slightly better in MB solution discoloration in comparison with the unmodified films, the composite films presented remarkable dye adsorption (almost twice) during the dark conditions, implying more available surface for dye adsorption and photodegradation. In the case of RhB, the improvement of the photocatalytic performance was clear for TCD. Τhe addition of carbon dots on the titania films increased the removal efficiencies from 69.68 to 79.78% for TCD, without affecting the dye adsorption (Table 4).

**Table 4:** The removal efficiency and rate constant of MB and RhB with the synthesized films

Samples	Removal efficiency (%)		Rate constant min^−1^	
	MB	RhB	MB	RhB
T	79.33	69.68	0.01414	0.00994
TCD	81.49	79.78	0.01406	0.01332

Regarding these results, the beneficial carbon dots incorporation with titania was evident, though there was no indication of how these materials interact. It is known that MB and RhB degradation with TiO_2_ nanomaterials under UV-A follows the oxidation pathways, in which hydroxyl radicals and the holes are the most effective species (Shammi et al. 2020; Mishra et al. 2019). The carbon-dot modification should suppress the photogenerated charge carrier recombination; hence, more holes should be available for the direct dye oxidation and the ^●^OH formation. However, indicative trap experiments revealed that only the h^+^ participate in the MB degradation (Fig. 11c). This trend shows that the quantum dots do not only improve the e^−^/h^+^ separation by acting as electronic sink for the photoelectrons, but also alter the MB degradation mechanism of TiO_2_ catalysts.

#### Reusability experiments

**Fig. 12:**
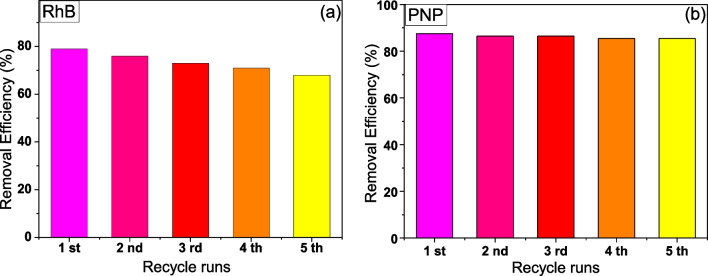
Photocatalytic oxidation of RhB performance (**a**) and photocatalytic reduction of PNP performance (**b**) with TCD under UV irradiation upon five consecutive recycling runs

In order to examine the applicability of the TCD films, the photocatalytic oxidation and reduction of RhB and PNP were repeated for five consecutive runs under the same photocatalytic experimental conditions, as presented in Fig. 12. Before each photocatalytic cycle, the nanocomposite films were first recovered through washing with DI H_2_O and dried in ambient conditions. The results showed that TCD displays an excellent activity during the reusability experiment, with a very small loss of its activity. Particularly, the degradation efficiency of RhB after the 5th cycle run was around 75%, close to the initial value of 79.78%, while the PNP reduction efficiency was maintained at about 85.5% after the fifth cycle. Moreover, the films were remained intact after the reusability experiments, without any deterioration for the materials, assuring their excellent and durable high stability during photocatalytic performance in both oxidation and reduction reaction pathways. Considering these results, TCD material possesses a high impact and potential for up-scaled water purification applications.

## Conclusion

Novel bi-functional photocatalytic films (C-QDs/TiO_2_) were prepared via microwave-assisted hydrothermal procedures and their morphology, crystallinity, and physicochemical properties were studied with microscopy and spectroscopy techniques. The carbon quantum dots incorporation with TiO_2_ materials was approved through FTIR and XPS spectroscopy. C-QDs improved significantly the composite’s optoelectronic properties, without causing any obvious alterations on TiO_2_’s structural properties. Organic and inorganic pollutants were chosen to evaluate the photocatalytic properties of the composites, where the composite quantum dot/titania films show improved photocatalytic activity, at least 10% improvement in comparison with the reference, for the degradation of MB and RhB dyes as well as the reduction of hexavalent chromium and PNP, even after 5 photocatalytic cycles. Scavenger experiments were performed to elucidate the reaction mechanism for both oxidation and reduction reaction, confirming the photogenerated holes (h^+^) as the main active species for oxidation reactions and the photoelectrons for reduction. Reusability tests were also accomplished, in order to confirm the stability and reusability of the composite films in long-term applications.

## Data Availability

All data generated or analyzed during this study are included in this published article.
